# Supplementary material for: MOFs-Derived Nano-CuO Modified Electrode as a Sensor for Determination of Hydrazine Hydrate in Aqueous Medium
Source: Sensors (Basel). 2019 Dec 24;20(1):140. doi: 10.3390/s20010140 (PMC6982735; doi:10.3390/s20010140)
Supplement: Supplementary file 1 [file sensors-20-00140-s001.pdf]

Supplementary Materials

MOFs-derived Nano-CuO Modified Electrode as a Sensor for Determination of Hydrazine Hydrate in Aqueous Medium

Yaqi Lu, Dan Wu, Ziyin Li,, Quanjie Lin, Xiuling Ma*, Zhangjing Zhang, and Shengchang Xiang*

College of Materials Science and Engineering, Fujian Provincial Key Laboratory of Polymer Materials, Fujian Normal University, 32 Shangsan Road, Fuzhou 350007, PR China

Synthesis of HKUST-1: HKUST-1 was synthesized by the solvothermal method [1]. $Cu(NO_3)_2 \cdot 3H_2O$ (1.7 mmol, 0.4154 g) was dissolved in 3 mL distilled water, and 1,3,5-Benzenetricarboxylic Acid (0.95 mmol, 0.2 g) was dissolved in 3 mL ethanol and 3 mL DMF. The two solutions were mixed and shaken and transferred to a transparent glass bottle and reacted at 85 °C for 12 h. The crystals were filtered, washed several times with acetone and dried under vacuum at 120 °C for 24 h.

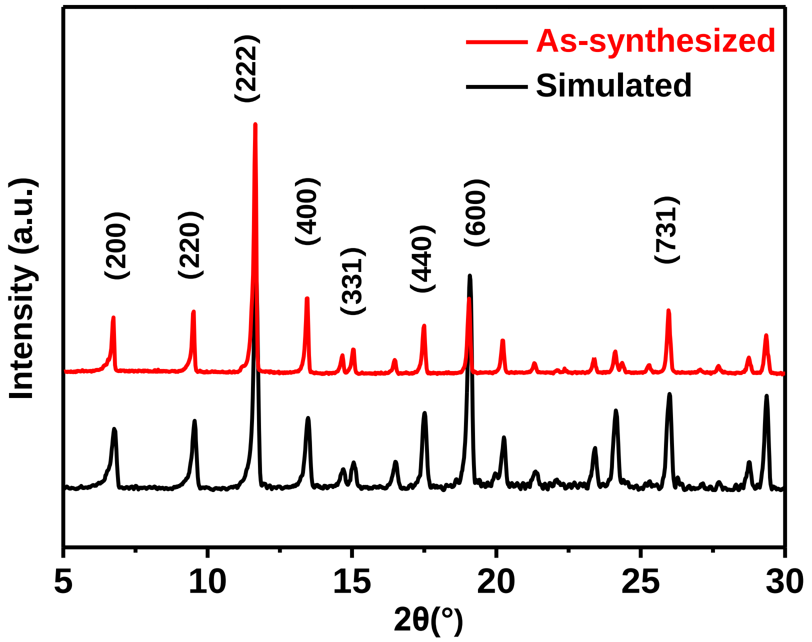

Figure S1: PXRD patterns of the simulated and synthesized HKUST-1.

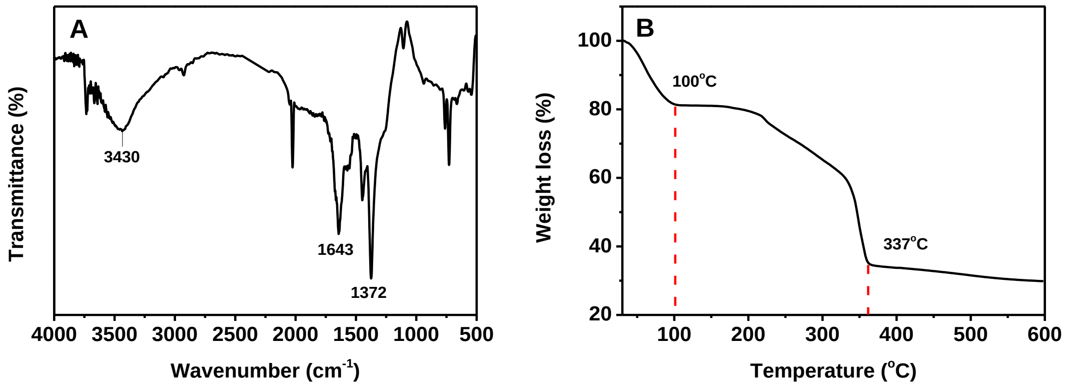

Figure S2: The FT-IR spectra (A)and TGA pattern (B) of HKUST-1

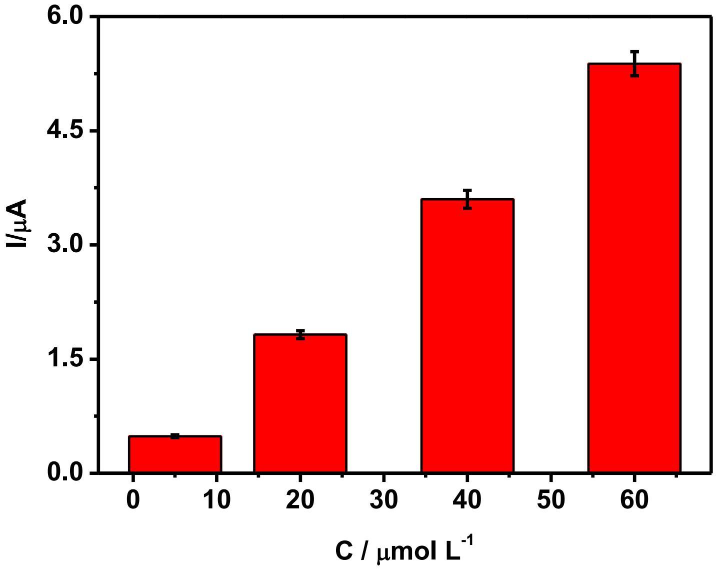

Figure S3: The amperometric response of spiked sample analysis

Table S1. Comparison for the determination of hydrazine at different modified electrodes.

Modified materials	Linear range (μmol L^{-1})	Detection limit (μmol L^{-1})	Reference
Pd-MWCNTs	0.1–10	0.016	[2]
Pt–TiO2	2–1030	0.142	[3]
Pd/carbon black	20–500	8.8	[4]
Cu–CuO	100–1800	—	[5]
CuS–rGO	1–1000	0.3	[6]
CuO hollow spheres	5–10000	1.9	[7]
Cu/Cu2O@carbon	0.25–800	0.022	[8]
Au-SH-SiO2@Cu-MOF	0.04–500	0.01	[9]
nano-copper oxide	0.1-600	0.03	[10]
MB-UiO-66-NH_2	—	0.012/0.37	[11]
GDC-NiO-300	100-991	1.5	[12]
Nano-CuO-400	1.98-169.3/232-2096	0.0295/0.0707	This work

Table S2. Results for determination of hydrazine in the water samples.

Sample	C_a(μmol/L)	$C_d{}^a$(μmol/L)	RSD(%)	Recovery(%)
1	5	5.16	3.87	103.2
2	20	19.74	2.89	98.7
3	40	41.12	3.26	102.8
4	60	61.25	2.96	102.1

a: Average of three determinations

References

[1] Xiang, S., Zhou, W., Gallegos, J. M., Liu, Y., and Chen, B., *Journal of the American Chemical Society*, 2010, vol. 131, no. 34, p. 12415.

[2] Haghighi, B., Hamidi, H., and Bozorgzadeh, S., A*nalytical & Bioanalytical Chemistry*, 2010, vol. 398, no. 3, p. 1411.

[3] Ding, Y., Wang, Y., Zhang, L., Zhang, H., Li, C. M., and Lei, Y., Nanoscale, 2011, vol. 3, no. 3, p. 1149.

[4] Panchompoo, J., Aldous, L., Downing, C., Crossley, A., and Compton, R. G., *Electroanalysis*, 2011, vol. 23, no. 7, p. 1568.

[5] Bassetto, V. C., Russell, A. E., Kubota, L. T., and Bartlett, P. N., *Electrochimica Acta*, 2014, vol. 144, p. 400.

[6] Yang, Y. J., Li, W., and Wu, X., *Electrochimica Acta*, 2014, vol. 123, p. 260.

[7] Khan, S. B., Faisal, M., Rahman, M. M., Abdel-Latif, I. A., Ismail, A. A., Akhtar, K., and Alamry, K. A., *New Journal of Chemistry*, 2013, vol. 37, no. 4, p. 1098.

[8] Zhao, Z., Wang, Y., Li, P., Sang, S., Zhang, W., Hu, J., and Lian, K., *Analytical Methods*, 2015, vol. 7, no. 21, p. 9040.

[9] Hosseini, H., Ahmar, H., Dehghani, A., Bagheri, A., Fakhari, A. R., and Amini, M. M., *Electrochimica Acta*, 2013,vol. 88, p. 301.

[10] Yin, Z., Liu, L., and Yang, Z., *Journal of Solid State Electrochemistry*, 2011,vol. 15, no. 4, p. 821.

[11] Helal, A., Qamaruddin, M., Aziz, M. A., Shaikh, M. N., and Yamani, Z. H., *ChemistrySelect*, 2017, vol. 2, no.25, p. 7630.

[12] Sivakumar M , Veeramani V , Chen S M , et al. *Microchimica Acta*, 2019, 186(2):59.
